# Papuamine causes autophagy following the reduction of cell survival through mitochondrial damage and JNK activation in MCF-7 human breast cancer cells

**DOI:** 10.3892/ijo.2013.2093

**Published:** 2013-09-10

**Authors:** SYU-ICHI KANNO, SHIN YOMOGIDA, AYAKO TOMIZAWA, HIROYUKI YAMAZAKI, KAZUYO UKAI, REMY E.P. MANGINDAAN, MICHIO NAMIKOSHI, MASAAKI ISHIKAWA

**Affiliations:** 1 Departments of Clinical Pharmacotherapeutics, Tohoku Pharmaceutical University, Aoba-ku, Sendai 981-8558, Japan;; 2 Natural Product Chemistry, Tohoku Pharmaceutical University, Aoba-ku, Sendai 981-8558, Japan;; 3 Faculty of Fisheries and Marine Science, Sam Ratulangi University, Kampus Bahu, Manado 95115, Indonesia

**Keywords:** papuamine, autophagy, cytotoxicity, mitochondrial damage, c-Jun N-terminal kinase

## Abstract

We previously reported that extracts of an Indonesian marine sponge *Haliclona* sp. showed potent cytotoxicity and the induction of apoptosis against human solid cancer cell lines. In this study, we examine the cytotoxic mechanism of the major chemical compound, papuamine, on MCF-7 human breast cancer cells. Papuamine at 5 *μ*M did not show significant cytotoxic effects after incubation for 24 h, but autophagosome vesicular formation was apparent. At 10 *μ*M of papuamine, significant reduction in cell survival was observed at 12 h, and increases in autophagy at this concentration were time-dependent and apparent before the appearance of cytotoxic effects. Both the release of cytochrome c to the cytosol and increase in Bax in the mitochondrial fraction were found to be concentration-dependent. Moreover, mitochondrial membrane potential shows concentration- and time-dependent decreases with exposure to papuamine. The release of cytochrome c has been shown to be accompanied by an increase in JNK activation. 3-Methyladenine (MA), a classical autophagy inhibitor showed increased JNK activation by exposure to papuamine. In conclusion, our results indicate that papuamine causes earlier onset autophagy and delayed reduction of cell survival through mitochondrial damage and JNK activation in MCF-7 cells.

## Introduction

Autophagy, a catabolic degradation process, has recently been identified as an important stress response and cell death regulatory mechanism (1). It is characterized by the formation of double-membrane autophagosomes, which enclose cytosolic materials and organelles, and then fuse with lysosomes, leading to the degradation of the luminal content by lysosomal proteases (2). Autophagy is also considered to be a cell survival pathway that plays roles in development, immunity (3) and cell death (4); it has been implicated in neurodegeneration, autoimmunity and cancer (1). Recent studies have reported the induction of autophagy at early stages after chemotherapeutic treatment (5,6) and that deregulation of its mechanisms contributes to therapeutic resistance (7,8).

Stress-induced apoptosis in mammalian cells often proceeds through the intrinsic pathway mediated by mitochondria. Once cells are committed to the apoptosis pathway, a number of downstream events are triggered that execute cell death, including permeabilization of the mitochondrial outer membrane, release of several apoptogenic mitochondrial proteins such as cytochrome c and activation of the caspase cascade (9,10). Many signaling pathways involved in the regulation of autophagy also regulate apoptosis. The molecular regulators of both pathways are interconnected; numerous death stimuli are capable of activating either pathway, and the pathways share several genes that are critical to their respective functions (11,12). Ham *et al* (13) identified the interrelationship between autophagy and apoptosis regulation by c-Jun N-terminal kinase (JNK) activation. Activation of JNK contributes to a change in the Bcl-2 family protein. Other reports indicate that mitochondrial Bcl-2 family proteins play important roles in the crosstalk between autophagy and apoptosis (14). The signals targeting individual mitochondria for autophagy are not known, but it is suggested that changes in mitochondria membrane potential are important events that provide such signals (15).

In 1988, Scheuer and co-workers reported the isolation of papuamine from *Haliclona* sp., a marine sponge collected at South Lion Island, Papua, New Guinea, and papuamine was demonstrated to inhibit the growth of the dermatophyte *Trichophyton mentagrophytes* (16). We previously reported that the extract of an Indonesian marine sponge *Haliclona* sp. showed potent cytotoxicity against the following human solid cancer cell lines (17): MCF-7 (breast), LNCap (prostate), Caco-2 (colon) and HCT-15 (colon). Studies on nuclear morphological changes and flow cytometric analysis suggested that an active component in the extract induced apoptosis in these cancer cells, and this major cytotoxic chemical compound was identified as papuamine.

In this study we examined the cytotoxic mechanism of papuamine on human breast cancer MCF-7 cells and clarified its involvement in autophagy and mitochondria damage. In particular, we focused on mitochondria dysfunction, changes in anti- or pro-apoptotic mitochondrial proteins, such as the Bcl-2 family, release of cytochrome c, and JNK activation by papuamine.

## Materials and methods

### Chemicals and cell cultures

Papuamine was isolated from Indonesian marine sponge *Haliclona* sp. by our previously published methods (17). Papuamine was dissolved in dimethyl sulfoxide (DMSO) and stored as a 20-mM stock solution in light-proof containers at −20°C. 3-Methyladenine (3-MA), and all other reagents, unless otherwise stated, were of the highest grade available and were supplied by either Sigma (St. Louis, MO, USA) or Wako Pure Chemical Industries, Ltd. (Osaka, Japan). Exposure to light was kept to a minimum for all drugs used. Human breast cancer MCF-7 cell line was supplied by the Cell Resource Center for Biomedical Research, Tohoku University (Sendai, Japan). Cells were maintained in RPMI-1640 medium supplemented with 10% fetal bovine serum, 100 U/ml penicillin G, and 100 *μ*g/ml streptomycin at 37°C in a humidified 5% CO_2_-95% air incubator under standard conditions. Cell counts were determined, excluding cells stained with 0.2% Trypan blue. To maintain exponential growth, cells were seeded at 5×10^4^ cells/ml and passaged every 3–4 days. Cells were cultured at 0.5×10^5^–1×10^5^ cells/ml in 100 *μ*l in separate wells of 96-well plates or in 2 ml aliquots in 35 mm dishes for other assays.

### Cytotoxicity

Cytotoxicity was assessed by the MTT [3-(4,5-dimethylthiazol-2-yl)-2,5-diphenyl tetrazolium bromide] assay described previously (18) but with modifications. Briefly, cells were incubated in 96-well plates with papuamine, followed by the addition of 10 *μ*l of MTT (5 mg/ml saline) to each well. The samples were incubated for 90 min at 37°C, the supernatant was aspirated, and the cells were lysed and solubilized by the addition of 100 *μ*l of 0.04 N HCl in isopropanol. The absorbance was measured for each well at 590 nm using a SH-1200 Microplate Reader^®^ (Corona, Hitachinaka, Japan). Control cells were treated with 0.1% DMSO. Cell viability was calculated by the following formula:

$$\text{Cell}\;\text{viability}\left(\%\right)=\frac{\text{Absorbance}\;\text{in}\;\text{treated}\;\text{sample}}{\text{Absorbance}\;\text{in}\;\text{control}}\times 100\%$$

### Confocal fluorescence microscopy for detection of protein 1 light chain 3 (LC3)

Cells were seeded into the Lab-Tek^®^ 8-well chambered cover glass system (Thermo Fisher Scientific, Inc., Rockford, IL, USA) at densities of 2×10^4^ and incubated overnight under standard culture conditions. Cells were then treated with papuamine at a dose of 0 (treatment with 0.1% DMSO as control group), 2.5, 5 or 10 *μ*M and incubated for an additional 24 h. The chambered slides were washed with phosphate buffered saline (PBS) twice and fixed in ice-cold 1:1 methanol:acetone for 30 min. The slides were immersed for 50 min in 1% goat serum and 0.1% Triton X-100 in PBS and then transferred to 10% goat serum/PBS for 20 min. Following the PBS rinse, slides were incubated with primary antibody (anti-LC3; MBL, Nagoya, Japan) at 1:1000 in PBS for 1 h at room temperature, washed with PBS, and then incubated with fluorescein isothiocynate (FITC)-conjugated anti-rabbit secondary antibody (Beckman Coulter, Brea, CA, USA) for 30 min. After rinsing with PBS and a drop of UltraCruz™ Mounting Medium with DAPI (Santa Cruz Biotechnology, Inc., Dallas, TX, USA) was added to each well. The cells were observed under a confocal fluorescence microscope C-1 (Nikon, Tokyo, Japan) and the blue fluorescence intensity (405 nm) for observation of nuclear or green fluorescence intensity (488 nm) to detect the LC3-positive cells that are indicative of autophagy. Image analysis software was used to ImageJ 1.46 supplied by National Institute of Health (NIH, Washington, DC, USA), and quantitative results were expressed as fluorescence intensity of LC3 at 488 nm.

### Mitochondrial membrane potential assay

Following the induction of permeability transition (19) using a TECAN infinite^®^ M1000 microplate reader (Tecan Group Ltd., Männedorf, Switzerland) at an excitation wavelength of 507 nm and an emission wavelength of 529 nm, mitochondrial membrane potential was estimated as the fluorescence of Rhodamine 123 (R123) staining. Briefly, cells were seeded at a density of 5×10^3^ in Nunc 96 MicroWell™ optical bottom plate (Thermo Fisher Scientific, Inc.) and incubated overnight under standard culture conditions. Media with various concentrations of papuamine was renewed and cultures were incubated for the indicated times. To conduct the assay, R123 at 0.1 *μ*M was added to the wells, the plates were allowed to sit for 15 min, the reaction mixture was removed, and fluorescence intensity was determined. The fluorescent intensity of each papuamine treatment group was compared to that of the control group in three independent experiments and calculated as a percent.

### Western blot analysis

Cells were washed with PBS and lysed in CelLytic M^®^ (Sigma) to collect a total cell lysate, or to separate between cytosol and mitochondria protein fractions were collected using the Mitochondria Isolation Kit^®^ (Sigma) according to the manufacturer’s instructions. Following electrophoreses of protein samples (10 *μ*g) of each protein on 7.5–12.5% SDS-polyacrylamide gels, the protein was transferred to a polyvinylidene difluoride (PVDF) membrane. Protein was blocked with Blocking One^®^ (Nacalai Tesque, Inc., Kyoto, Japan) for 1 h and incubated with antibody overnight at 4°C. The membrane was then washed with wash buffer (PBS containing 0.05% Tween-20) and incubated with horseradish peroxidase-linked secondary antibody for 1 h. After another wash with wash buffer, protein levels were analyzed by enhanced chemiluminescence with Pierce^®^ Western Blotting substrate (Thermo Fisher Scientific, Inc.).

### Statistical analysis

Statistical analysis was performed by one- or two-way analysis of variance (ANOVA) followed by Williams’ type multiple comparison test or Bonferroni test among multiple groups. Data are expressed as mean ± standard deviation (SD). A p-value of less than 0.05 was considered to be statistically significant.

## Results

### Cytotoxic effects of papuamine on human breast cancer MCF-7 cells

**Figure 1.**
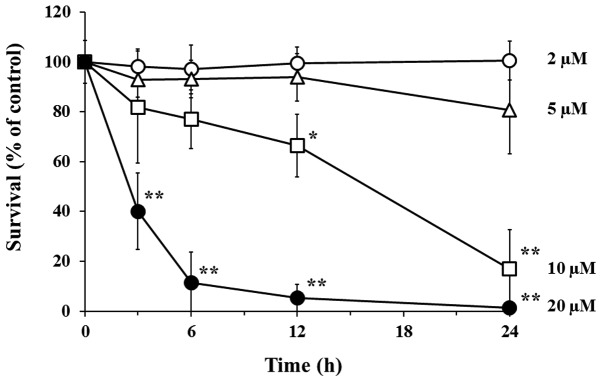
Papuamine-induced reduction of cell survival in human breast cancer MCF-7 cells. Cells were incubated with the indicated concentrations of papuamine for the indicated durations, following which cell survival was assayed using the MTT assay. Survival (%) was calculated relative to the control (Papuamine vehicle). The results are means ± SD of three individual studies. ^*^p<0.05, ^**^p<0.01 compared with control.

Examination of the cytotoxic effects of papuamine on human breast cancer MCF-7 cells cultured for 24 h by MTT assay showed that there were no cytotoxic effects with 2 *μ*M (Fig. 1). Incubation with 5 *μ*M papuamine showed slightly decreased cell survival (80.6±17.6%) at 24 h, but the decease was not significant. Incubation with 10 *μ*M papuamine at 12 or 24 h resulted in significantly decreased cell survival at 66.4±12.6% (p<0.05) or 16.9±15.7% (p<0.01), respectively. Incubation with higher concentration of papuamine (20 *μ*M) at 3, 6, 12 and 24 h showed significantly decreased cell survival to 40.0±15.3%, 11.3±10.4%, 5.2±2.7% and 1.3±1.1%, respectively (p<0.01). Thus, papuamine showed time- and concentration-dependent cytotoxic effects on MCF-7 cells.

### Detection of papuamine-induced autophagy by confocal fluorescence microscopy

**Figure 2.**
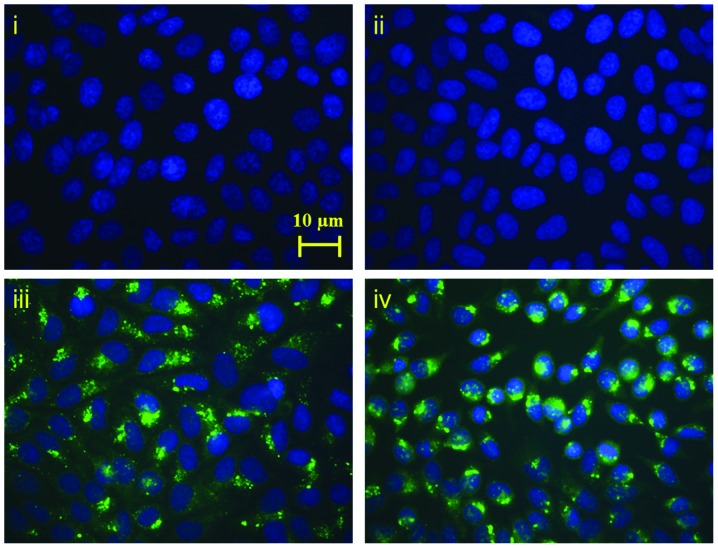
Confocal fluorescence microscopy for LC3 detection in papuamine-treated MCF-7 cells. Cells were incubated for 24 h with 2.5 (ii), 5 (iii), or 10 *μ*M papuamine (iv) and compared with untreated controls (i). Images were co-stained with nuclear dye DAPI (magnification, ×400).

**Figure 3.**
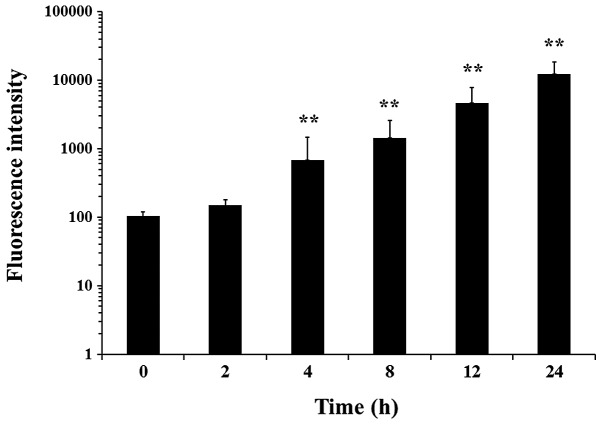
The effect of time-dependent quantitative fluorescence intensity of LC3 on 10 *μ*M papuamine-treated MCF-7 cells. Data are shown as mean ± SD values from three independent experiments. ^**^p<0.01 compared with control.

To examine the cytotoxic mechanism of papuamine related to the induction of autophagy, we detected protein 1 LC3, a hallmark of mammalian autophagy, by confocal fluorescence microscopy (Fig. 2). LC3 is essential for the formation of autophagosomes and for the completion of macroautophagy (20). The control group and 2.5 *μ*M papuamine group MCF-7 cells showed no evidence of LC3 expression, but LC3 was observed in cells treated with papuamine at 5 *μ*M or greater, as indicated by heavy (green) staining that reveals clear autophagosome vesicle formation. We conducted quantitative analysis of 10 *μ*M papuamine-induced autophagy on the abundance of LC3-positive staining using an image analyzer (Fig. 3). The fluorescence intensity of LC3 showed little change with papuamine incubation for 2 h, but significant time-dependent increase was observed for incubation for 4 h (p<0.01).

### Change in mitochondrial proteins and mitochondria membrane potential with papuamine exposure

**Figure 4.**
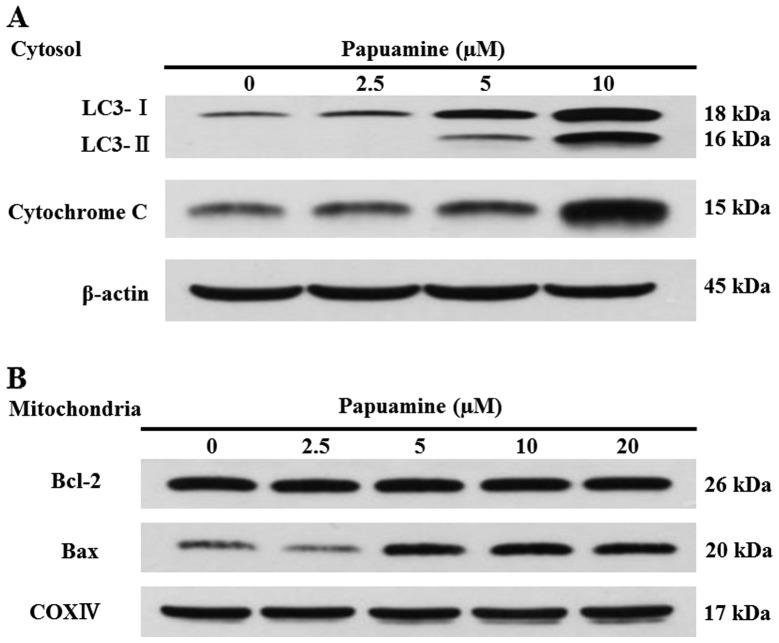
Western blot analysis the release of cytochrome c to cytosol and change of Bcl-2 or Bax mitochondria protein by incubation with papuamine for 24 h. Cells were incubated with the indicated concentration of papuamine for 24 h, and samples were prepared as described in Materials and methods. Expression of the indicated proteins was analyzed by western blot analysis using expression of β-actin for cytosol fraction (A) or COXIV for mitochondria fraction (B) as loading controls, respectively. Experiments shown are representative of a minimum of three independent experiments.

**Figure 5.**
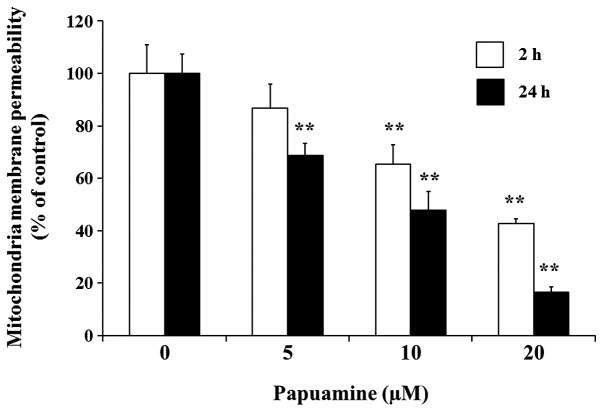
Effects of papuamine on mitochondrial membrane potential. Incubation with papuamine at indicated concentrations for 2 or 24 h, the mitochondrial membrane potential was analyzed using fluorescence microplate reader with Rhodamine 123 staining. Results are presented as the mean ± SD from three independent experiments. ^**^p<0.01 compared with control.

To determine whether papuamine regulates autophagy, we examined the rate of conversion of LC3-I to LC3-II via proteolytic cleavage and lipidation. LC3-II is an LC3-phosphatidylethanolamine conjugate and a promising autophagosomal marker (21). Papuamine at 2.5–10 *μ*M showed concentration-dependent increases in the lipidated form of LC3 (LC3-II) with 24 h incubation (Fig. 4A). To explore the effect of papuamine on mitochondrial protein, we examined the release of cytochrome c to the cytosol (Fig. 4A) and the changes of Bcl-2 and Bax proteins in the mitochondria fraction (Fig. 4B). The loading control of cytosol or mitochondria fractions for β-actin or cyclooxygenase IV (COXIV), respectively, was not changed in the experimental conditions. Both the release of cytochrome c to cytosol and change of Bax in the mitochondria fraction showed increases that were papuamine-dependent, but Bcl-2 expression did not change. To confirm the effect of papuamine on the mitochondrial membrane potential of MCF-7 cells, we examined R123 fluorescence (Fig. 5). In incubation with papuamine at 5, 10 and 20 *μ*M for 2 h, mitochondria membrane permeability showed a concentration-dependent decrease to 86.7±9.3%, 65.3±7.4% (p<0.01) and 42.8±1.8% (p<0.01), respectively. These effects were significantly enhanced (p<0.01) with longer incubation (24 h) to 68.7±4.6% with 5 *μ*M, 47.9±6.9% with 10 *μ*M and 16.5±1.9% with 20 *μ*M. Thus, mitochondrial membrane potential shows concentration- and time-dependent decreases with exposure to papuamine.

### Detection of time-dependent JNK activation and release of cytochrome c or effects of 3-MA, an autophagy inhibitor on papuamine-induced autophagy

**Figure 6.**
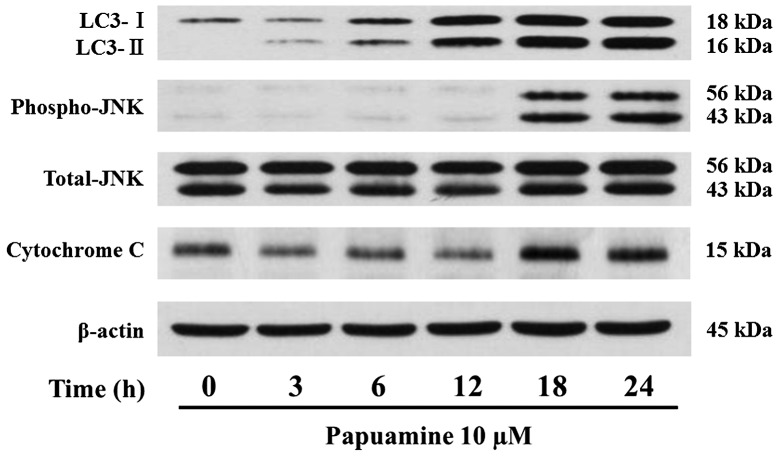
Time-dependent western blot analysis of proteolytic LC3, JNK activation and cytochrome c by incubation with papuamine at 10 *μ*M. Cells were incubated with 10 *μ*M papuamine for the indicated times, and samples were prepared as described in Materials and methods. The expression of the indicated proteins was analyzed by western blot analysis using the expression of β-actin as a loading control. Data shown are representative of a minimum of three independent experiments.

**Figure 7.**
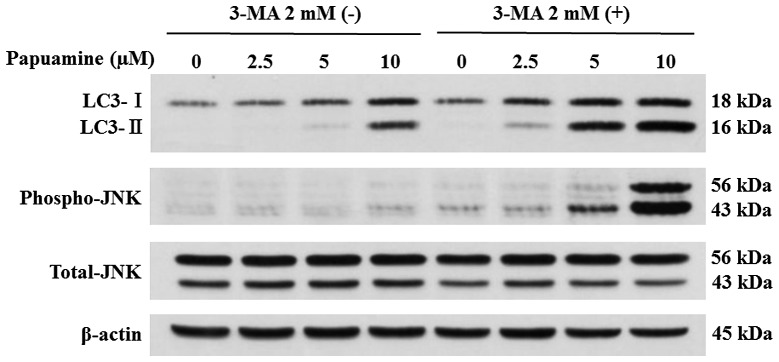
The effects of 3-MA on papuamine-induced proteolytic LC3, or JNK activation by western blot analysis. Pretreatment with 2 mM of 3-MA incubation for 4 h and the following incubation with the indicated concentration of papuamine for 12 h. The expression of the indicated proteins was analyzed by western blot analysis using expression of β-actin as a loading control. Experiments shown are representative of a minimum of three independent experiments.

To ascertain whether papuamine-induced autophagy is related to JNK activation, we examined the time-dependent effects of 10 *μ*M papuamine over 24 h incubation (Fig. 6). The proteolytic LC3 increased gradually from early stages of the incubation with papuamine. The phosphorylation of JNK, an activated form was barely present with incubation up to 12 h, but was observed at higher concentrations at 18 and 24 h. The release of cytochrome c mirrored the increase in JNK activation. 3-MA has been identified as a classical autophagy inhibitor (22) and has been widely used as a pharmacological tool in the studies of autophagy. 3-MA at 2 mM had no cytotoxic effects on MCF-7. In the incubation for 12 h with papuamine alone at 0–10 *μ*M, proteolytic LC3 showed a concentration-dependent increase, but JNK activation was not detected (Fig. 7). JNK activated LC3 lipidation was apparently increased by pretreatment with 3-MA. Both the expression of total JNK and β-actin as loading controls were nearly constant for all treatment groups in this study.

## Discussion

Our previous study indicated that the extract of an Indonesian marine sponge *Haliclona* sp., which has papuamine as a major constituent, exhibited cytotoxicity and induced apoptosis in human solid cancer cell lines (17). In this study, we demonstrated that papuamine cytotoxicity to human breast cancer MCF-7 cells is attributable to the induction of autophagy. The relationship between apoptosis and autophagy has been widely studied. According to Jia *et al* (23), autophagy may promote apoptosis in some systems. It was also reported that autophagy occurs earlier than apoptosis (24,25); however, autophagy is probably not involved in the death process unless apoptosis is blocked (26). These cells preferentially die by apoptosis, but in the absence of apoptosis, they will die by any alternative available route, including autophagy (27). It is possible that the effect of autophagy on apoptosis is cell line- and stimulus-dependent.

As shown in Fig. 1, papuamine at 5 *μ*M does not show significant cytotoxic effects in 24 h incubation, but autophagosome vesicular formation is marked at this time point (Fig. 2). With a higher concentration of papuamine (10 *μ*M), cells showed significant reduction in survival at 12 h (Fig. 1) and concentration- and time-dependent increases in both LC3 (Fig. 3) and proteolytic LC3 (Fig. 6); these increases were evident prior to the observation of cytotoxic effects. Thus, the cytotoxic effect of papuamine was caused by the induction of autophagy in MCF-7 cells. A growing number of functional studies support the use of autophagy regulation in combination with established therapies for breast cancer treatment for improved clinical outcome (28). Since the early days of autophagy research, the anti-estrogen tamoxifen has been known to be a potent inducer of autophagy in a variety of breast cancer cells (29). We expect that papuamine will be added to the treatment regimen for breast cancer as a single agent or as a co-administration with chemotherapeutic agents such as tamoxifen.

Mitochondria are important intracellular organelles for producing energy from adenosine 5′-triphosphate (ATP), and dysfunction induced by DNA damage and other genotoxic factors leads to an irreversible event, cell death (30). As shown in Fig. 4, papuamine concentration-dependently increased both the release of cytochrome c to the cytosol and a change in Bax on the mitochondria fraction in MCF-7 cells. In this study, mitochondrial Bcl-2 expression was not affected by papuamine (Fig. 4B). It has been indicated that Bcl-2 does not protect against autophagic death (31). In contrast, Pattingre and Levine reported that Bcl-2 can protect against starvation-induced autophagy (32). This difference may arise from different stimuli. The mechanisms affecting Bcl-2 expression by pupamine in the present study have not been clarified, but earlier studies have indicated that an imbalance in the Bax/Bcl-2 ratio can render sensitivity to a wide variety of cell death stimuli, including all chemotherapeutic drugs, radiation, hypoxia, and growth factor withdrawal (33). Mitochondria dysfunction induces Bax translocation from the cytosol to the mitochondria, and cytochrome c release from the mitochondria is a critical event that occurs during the apoptotic processes (34). Motyl *et al* suggested that blocking caspases does not prevent Bax-induced cell death, as autophagic cell death is then initiated (35). The presence of Bax at the surface of mitochondria suggests a role for this organelle in autophagic cell death. Cytochrome c is normally found in the mitochondrial intermembrane space. Release of cytochrome c is most likely due to a decrease in mitochondria membrane potential. As shown in Fig. 5, the decrease in mitochondrial membrane potential was a result of time- and concentration-dependent exposure to papuamine. These results suggest that papuamine predominantly impairs the mitochondria. Therefore, elimination of damaged mitochondria may be critical to protect cells from apoptosis-promoting molecules released by dysfunctional mitochondria.

As shown in Fig. 6, the increase in proteolytic LC3 precedes both JNK activation and the release of cytochrome c with exposure to papuamine. Autophagy and apoptosis are fundamental cellular pathways, and are both regulated by JNK activation (13). Up-regulation of JNK triggers the release of mitochondrial cytochrome c, and activates the intrinsic death pathway (36). Lemasters *et al* (15) suggest that after autophagic stimulation, the change of mitochondria membrane potential appears to initiate mitochondrial depolarization and subsequent sequestration into autophagosomes. Moreover, autophagy occurring subsequent to cytochrome c release is likely to be triggered by mitochondrial outer membrane permeabilization and is therefore mitophagy, a recycling process by which mitochondria are captured and degraded (37). Our results corroborate the findings of these reports, namely, papuamine caused mitochondria damage and the induction of autophagy in early stages of exposure, and then these cellular events contribute to JNK activation and the release of cytochrome c, resulting in the reduction of cell survival accompanied by apoptotic cell death. JNK activation is known to regulate by allowing increased in Bax expression, which in turn leads to the execution of apoptosis (38). We considered that activation of JNK by papuamine emerges from increased Bax expression (Fig. 4B), and contribute to apoptotic cell death. Contrary to our expectation, inhibiting autophagy by pretreatment with 3-MA accelerated papuamine-induced autophagy and JNK activation (Fig. 7). Recent research showed that 3-MA was not a specific autophagy inhibitor (39), as it also inhibited phosphatidylinositol-3 kinase (40) and stimulated cAMP-dependent protein kinase (41). Presumably, papuamine has a pathway that not only has an inhibitory effect on 3-MA but also possesses a multiple cellular signal transduction to induce autophagy by pathways such as JNK activation or mitochondria damage. Inhibition the cellular pathway by chemical inhibitors likely induces other compensatory regulations.

In conclusion, our results indicate that papuamine causes early autophagy in human breast cancer MCF-7 cells following the late reduction of cell survival through mitochondria damage and JNK activation. The precise mechanisms underlying the role of autophagy in papuamine-induced cell death remain to be elucidated.
